# Cigarette smoke-induced cell death of a spermatocyte cell line can be prevented by inactivating the Aryl hydrocarbon receptor

**DOI:** 10.1038/cddiscovery.2015.50

**Published:** 2015-10-26

**Authors:** P Esakky, D A Hansen, A M Drury, A Cusumano, K H Moley

**Affiliations:** 1 Department of Veterans Affairs Medical Center, Washington University School of Medicine, St. Louis, MO, USA; 2 Department of Obstetrics and Gynecology, Washington University School of Medicine, St. Louis, MO, USA

## Abstract

Cigarette smoke exposure causes germ cell death during spermatogenesis. Our earlier studies demonstrated that cigarette smoke condensate (CSC) causes spermatocyte cell death *in vivo* and growth arrest of the mouse spermatocyte cell line (GC-2spd(ts)) *in vitro* via the aryl hydrocarbon receptor (AHR). We hypothesize here that inactivation of AHR could prevent the CSC-induced cell death in spermatocytes. We demonstrate that CSC exposure generates oxidative stress, which differentially regulates mitochondrial apoptosis in GC-2spd(ts) and wild type (WT) and AHR knockout (AHR-KO) mouse embryonic fibroblasts (MEFs). SiRNA-mediated silencing of *Ahr* augments the extent of CSC-mediated cellular damage while complementing the AHR-knockout condition. Pharmacological inhibition using the AHR-antagonist (CH223191) modulates the CSC-altered expression of apoptotic proteins and significantly abrogates DNA fragmentation though the cleavage of PARP appears AHR independent. Pretreatment with CH223191 at concentrations above 50 *μ*M significantly prevents the CSC-induced activation of caspase-3/7 and externalization of phosphatidylserine in the plasma membrane. However, MAPK inhibitors alone or together with CH223191 could not prevent the membrane damage upon CSC addition and the caspase-3/7 activation and membrane damage in AHR-deficient MEF indicates the interplay of multiple cell signaling and cytoprotective ability of AHR. Thus the data obtained on one hand signifies the protective role of AHR in maintaining normal cellular homeostasis and the other, could be a potential prophylactic therapeutic target to promote cell survival and growth under cigarette smoke exposed environment by receptor antagonism via CH223191-like mechanism. Antagonist-mediated inactivation of the aryl hydrocarbon receptor blocks downstream events leading to cigarette smoke-induced cell death of a spermatocyte cell line.

## Introduction

Habitual smokers have 13% lower sperm count than non-smokers.^1^ This is because the 7000 constituents, including 69 proven carcinogens, of cigarette smoke (CS) accumulate in the systemic circulation and seminal plasma and irreversibly impair both the quality and quantity of human spermatozoa.^2,3^ Chronic exposure to CS is toxic to germ cells^4,5^ and results in excessive generation of free oxygen radicals.^6^ Thus, the spermatozoa of cigarette smokers have increased levels of oxidative DNA damage and adducts, chromosomal abnormalities, and oxidized unsaturated fatty acids.^7^

Cigarette smoke condensate (CSC), or tar, consists mainly of dioxins, halogenated and nonhalogenated polycyclic aromatic hydrocarbons (PAHs), and pro-oxidants,^8^ which strongly inhibit meiotic progression of spermatocytes^9^ and stimulate genes involved in the metabolism of PAHs.^10^ PAHs and the dioxin 2,3,7,8-Tetrachlorodibenzo*-p-*dioxin (TCDD) act through the ligand-dependent transcription factor, aryl hydrocarbon receptor (AHR).^11^ Ge *et al.* ^12^ suggested that PAHs could impair gametogenesis by AHR-mediated suppression of meiosis. In addition, we demonstrated that *in vivo* exposure to CSC results in spermatocyte cell death and seminiferous tubule disruption.^13^ Furthermore, we reported that AHR is needed for proper seminiferous tubule architecture and sperm development.^14^ However, the role of AHR in apoptosis is unclear; some studies have indicated that AHR activation increases apoptosis, whereas others suggest that it decreases apoptosis.^15,16^ Many of these studies have relied on exogenously activating AHR with TCDD^17^ instead of the complex chemicals found in CS. Nonetheless, studies using AHR-knockout mice indicated that most of the TCDD-induced toxicity is mediated through AHR.^18^

As the mechanistic outcome of exposure to CSC is growth arrest followed by cell death in both *in vitro* and *in vivo* spermatocytes as demonstrated by our previous studies and to address the role of AHR in this process, we turned to the spermatocyte cell line GC-2spd(ts). We earlier found that CSC exposure altered the growth of spermatocytes by facilitating a crosstalk between MAPK and AHR-NRF2 pathways.^19^ Here we report that CSC promotes a mitochondrial-based apoptotic pathway in spermatocytes *in vitro*. CSC-exposed GC-2spd(ts) cells exhibited characteristic features of apoptosis such as altered expression of apoptotic proteins, DNA fragmentation, cleavage of Poly(ADP-ribose) polymerase, activation of executor caspases and externalization of phosphatidylserine on the membrane surface. We found that treating the spermatocytes with the AHR-specific inhibitor CH223191 significantly blunted the proapoptotic effect of CSC. However, knockdown of *Ahr* accompanied with enhanced CSC-mediated apoptosis indicates its endogenous protective role in maintaining tissue homeostasis. Our results provide evidence that development of an AHR inhibitor similar to CH223191 might provide a useful prophylactic to prevent the complications of exposure to CS and other similar pollutants.

## Results

### Cigarette smoke condensate creates oxidative stress in the spermatocyte cell line GC-2spd(ts)

**Figure 1:**
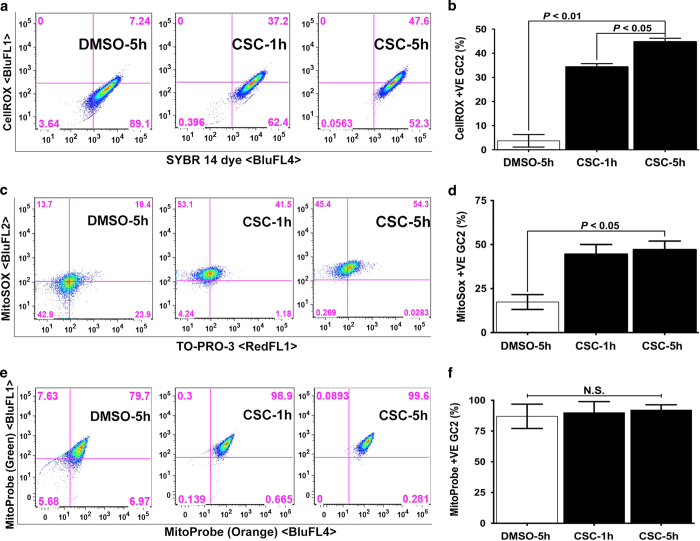
CSC induces oxidative stress but does not alter mitochondrial membrane potential in spermatocytes. (**a**, **c**, and **e**) Representative flow cytometric analyses of spermatocytes exposed to DMSO (0.1% for 5 h) or CSC (40 *μ*g/ml) for 1 and 5 h, then stained with (**a**) cellROX deep red reagent (BluFL1) and counter-stained with viable dye SYBR 14 (BluFL4), (**c**) mitoSOX superoxide indicator (BluFL1) and counter-stained with viable nuclear dye TO-PRO-3 (RedFL1), or (**e**) mitoProbe (Green, BluFl1 on *y* axis; Orange, BluFL4 on *x* axis). Percentages of double-positive cells are indicated in the upper right quadrants. (**b**, **d** and **f**). Histograms present the mean percentages of double-positive spermatocytes from three independent experiments, each assayed in triplicate,±S.E.M.

We previously used microscopy to demonstrate that GC-2spd(ts) cells (hereafter referred to as spermatocytes) accumulate reactive oxygen species after six hours of CSC exposure.^13^ To better quantitate this effect, we used flow cytometry to assess the percentage of cells that stained with cellROX, an indicator of cytoplasmic oxidative stress. We found that the percentage of cellROX-positive cells increased significantly upon exposure to 40 *μ*g/ml CSC (Figures 1a and b). In addition, flow cytometric analysis revealed that the percentage of cells positive for the mitochondrial superoxide indicator mitoSOX increased significantly upon CSC exposure (Figures 1c and d). We next assessed mitochondrial membrane potential by staining control and CSC-exposed cells with MitoProbe DiOC2(3). As a monomer, DiOC2(3) emits green fluorescence and, in a reaction driven by the mitochondrial membrane potential, converts to a red-fluorescence-emitting dimer. Here we found that CSC did not alter the membrane potential of mitochondria either at one or five hours of exposure (Figures 1e and f). Thus, CSC at 40 *μ*g/ml induces oxidative stress in both the cytoplasm and mitochondria of spermatocytes, but does not alter mitochondrial membrane potential.

### CSC-altered expression of BCL2 family members in spermatocytes is independent of AHR.

**Figure 2:**
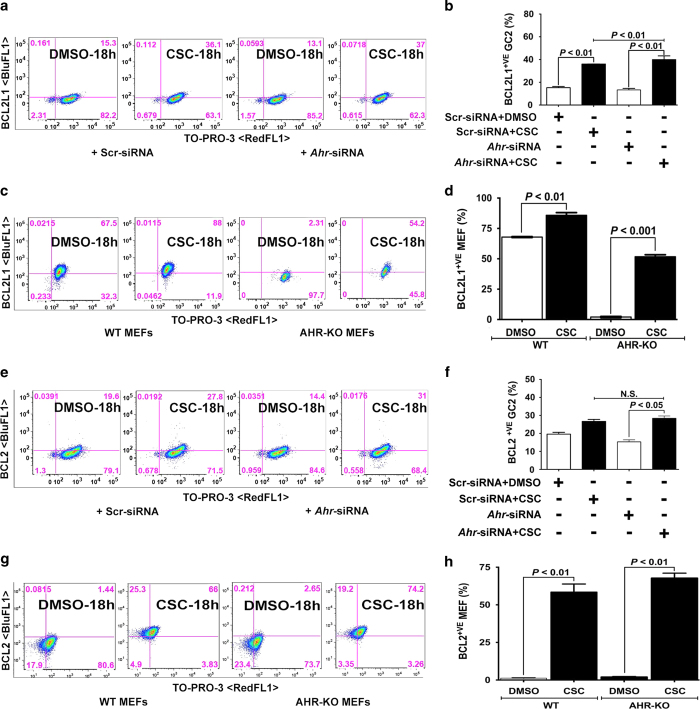
CSC modulates the expression of antiapoptotic proteins. (**a**, **c**, **e**, and **g**) Representative flow cytometric analyses of (**a** and **e**) spermatocytes transfected with scr-siRNA or *Ahr*-siRNAs and (**c** and **g**) WT and AHR-KO MEF cells treated with DMSO or CSC for 18 h and then stained with (**a** and **c**) anti-BCL2L1 (BluFL1) or (**e** and **g**) anti-BCL2 (BluFL1) antibody and counter-stained with TO-PRO-3 (RedFL1). Percentages of double-positive cells are indicated in the upper right quadrants. (**b**, **d**, **f** and **h**). Histograms present the mean percentages of double-positive spermatocytes and MEFs from three independent experiments, each assayed in triplicate,±S.E.M.

**Figure 3:**
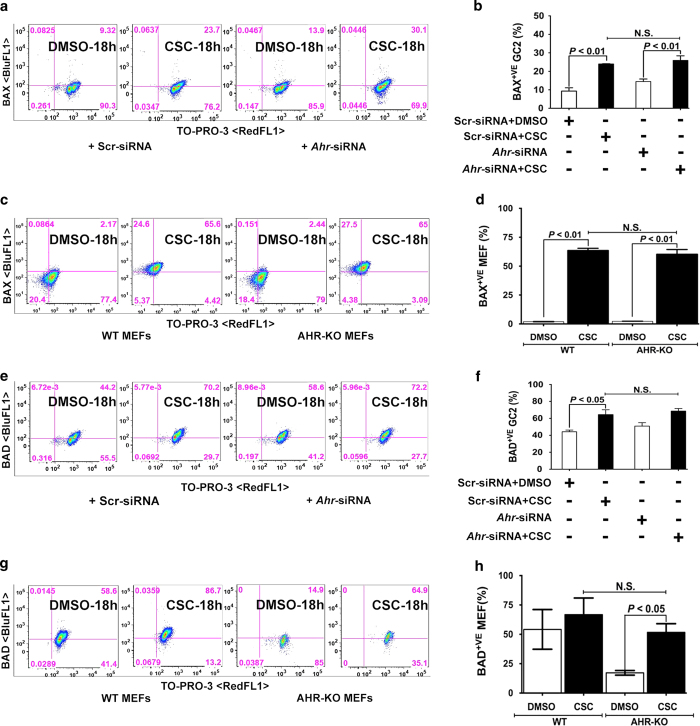
Regulation of proapoptotic proteins by CSC. (**a**, **c**, **e**, and **g**) Representative flow cytometric analyses of (**a** and **e**) spermatocytes transfected with scr-siRNA or *Ahr*-siRNAs and (**c** and **g**) WT and AHR-KO MEF cells treated with DMSO or CSC for 18 h and then stained with (**a** and **c**) anti-BAX (BluFL1) or (**e** and **g**) anti-BAD (BluFL1) antibody and counter-stained with TO-PRO-3 (RedFL1). Percentages of double-positive cells are indicated in the upper right quadrants. (**b**, **d**, **f** and **h**). Histograms present the mean percentages of double-positive spermatocytes and MEFs from three independent experiments, each assayed in triplicate,±S.E.M.

We next wanted to determine whether CSC exposure affects expression of apoptosis regulators in spermatocytes. Thus, we used flow cytometry to assess expression of the antiapoptotic proteins BCL2 and BCL2L1 and the proapoptotic proteins BAX and BAD. We found that exposure to CSC increased the percentage of spermatocytes expressing BCL2L1 (Figures 2a and b), BCL2 (Figures 2e and f), BAX (Figures 3a and b), and BAD (Figures 3e and f). To determine whether these changes require AHR, we evaluated *Ahr*-knock down spermatocytes by siRNA and treated them with CSC. Western blot showed that siRNA suppression has significantly (>70%) abrogated AHR expression in the spermatocytes (data not shown). However, we noticed that the *Ahr* knockdown did not prevent any of the CSC-induced gene expression changes in spermatocytes. Because siRNA-mediated knockdown is transient and or *Ahr* may be incompletely inactivated, we compared the effects of CSC exposure with another different cell type, the mouse embryonic fibroblasts (MEFs) isolated from wild type (WT) and *Ahr*-knockout (AHR-KO) mice. As in spermatocytes, we found that in MEFs, *Ahr* was not required for changes in the percentage of cells positive for BCL2L1, BCL2, BAX, and BAD upon CSC exposure (Figures 2c, d, g, h and 3c, d, g, h). These results suggest that CSC-induced oxidative stress activates the mitochondrial pathway of apoptosis in spermatocytes by differentially modifying the expression of apoptotic proteins in an AHR-independent manner.

### CSC exposure breaks DNA strands in spermatocytes.

**Figure 4:**
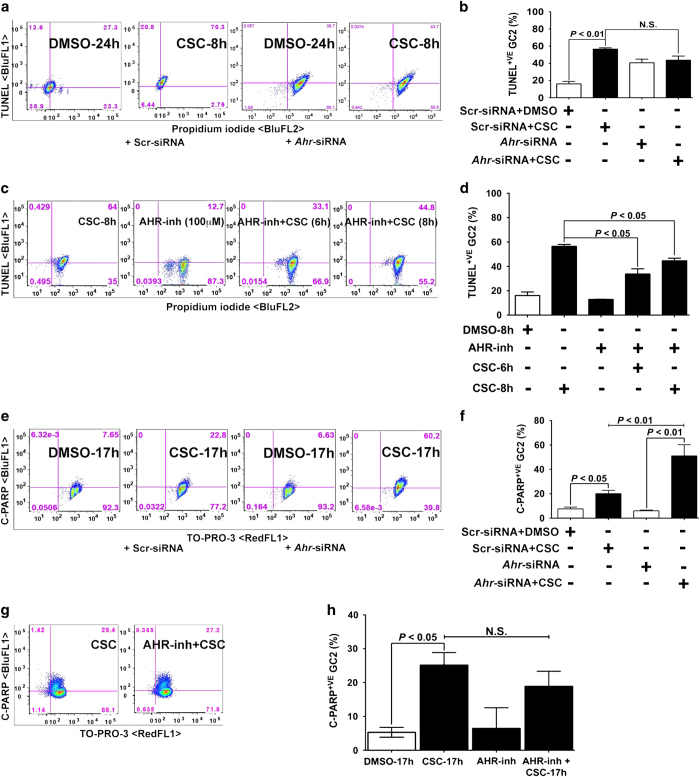
CSC exposure causes DNA fragmentation and cleavage of PARP in spermatocytes. (**a** and **e**) Representative flow cytometric analyses of spermatocytes transfected with scr-siRNA or *Ahr*-siRNAs and then treated with DMSO or CSC (40 *μ*g/ml) for (**a**) 8 h before TUNEL (BluFL1) and propidium iodide staining (BluFL2) or (**e**) 17 h before staining with anti-cleaved PARP antibody (BluFL1) and TO-PRO-3 (RedFL1). (**c** and **g**) Representative flow cytometric analyses of spermatocytes treated with CSC, AHR-inh, or both for 8 h before (**c**) TUNEL (BlurFL1) and propidium iodide staining (BluFL2) or (**g**) staining with anti-cleaved PARP antibody (BluFL1) and TO-PRO-3 (RedFL1). Percentages of double-positive cells are indicated in the upper right quadrants. (**b**, **d**, **f**, and **h**) Histograms present the mean percentages of double-positive spermatocyte from three or more experiments, each assayed in triplicate,±S.E.M.

Our previous studies showed that CSC exposure increased spermatocyte DNA damage *in vivo* and elevated the expression of genes associated with DNA damage *in vitro*.^13,19^ To determine whether CSC reflects similar effects in the spermatocyte cell line and to assess the role of AHR, we performed terminal deoxynucleotidyl transferase dUTP nick end labeling (TUNEL) in CSC-exposed spermatocytes. As expected, CSC significantly increased the number of TUNEL-positive cells. Knockdown of *Ahr* expression also increased DNA damage, but *Ahr* knockdown and CSC exposure together were not additive (Figures 4a and b). However, pretreatment with the AHR antagonist (CH223191, herein referred to as AHR-inh) significantly reduced the CSC-mediated increase in TUNEL-positive cells (Figures 4c and d). These data indicate that, although CSC-mediated DNA damage occurred in the absence of AHR, blocking AHR activation with an inhibitor blunted CSC-induced DNA damage.

Because poly(ADP-ribose) polymerase (PARP) is involved in DNA repair and is activated in response to DNA damage, we measured cleaved PARP-expressing cells by flow cytometry. We found that CSC exposure increased the percentage of spermatocytes expressing cleaved PARP by a moderate but significant amount. When *Ahr* expression was knocked down, an even higher percentage of CSC-treated spermatocytes expressed cleaved PARP (Figures 4e and f). However, we found an equal percentage of cleaved-PARP-expressing cells in the CSC and CSC plus AHR-KO groups (Figures 4g and h). We conclude that loss of *Ahr* exacerbated the CSC-mediated increase in the number of cleaved PARP-expressing spermatocytes but that inhibiting AHR neither ameliorated nor exacerbated the CSC-mediated increase in cleaved PARP-expressing cells.

### Is CSC-mediated activation of caspase-3/7 requiring AHR?

**Figure 5:**
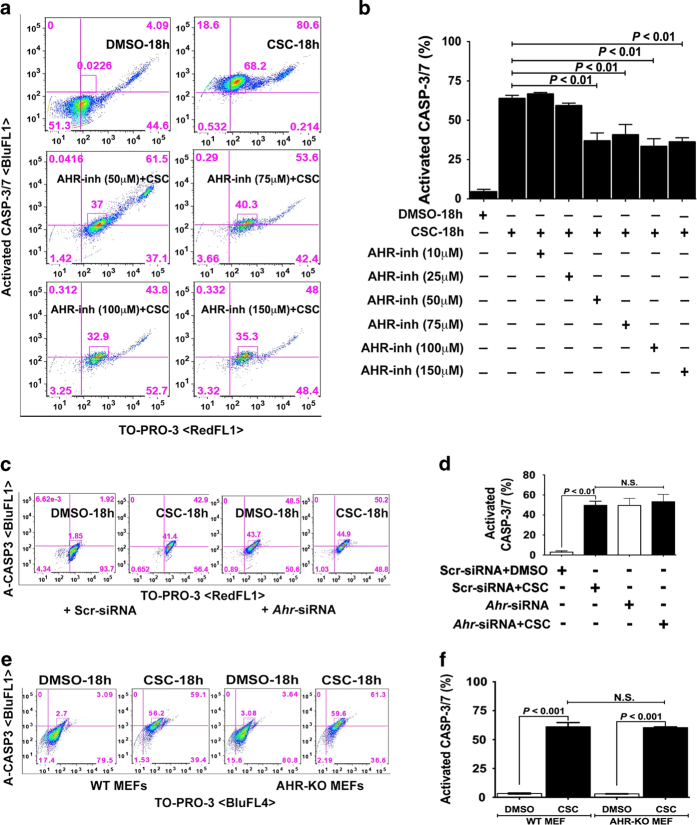
CSC modulated AHR mediates caspase-3/7 activation. (**a**, **c** and **e**). The flow cytometric data represent the distribution of GC-2spd(ts) cells (**a**) treated with DMSO or CSC (40 *μ*g/ml) for 18 h, AHR-inh alone at different concentrations (25, 50, 75, 100 and 150 *μ*M) or AHR-inh for 1 h followed by CSC, spermatocytes transfected with scr-siRNA or *Ahr*-siRNAs and (**c**) WT and AHR-KO MEF cells (**e**) treated with DMSO or CSC for 18 h. The treated spermatocytes were labeled by using CellEvent caspase-3/7 green detection reagent for assessing caspase activation and counter-stained with TO-PRO-3. The cell specimens of various groups were evaluated for positive cell populations based on standard compensation calculations on a BD FACSCalibur using FlowJo software (v9.7.5, FLOWJO, LLC., Ashland, OR, USA). The percent difference in caspase-3/7^+^ cells is shown within the quadrant. **b**, **d** and **f**). Histogram represents the mean flow cytometric data of percent of caspase-3/7^+^ (BluFL1 and RedFL1 double-positive) spermatocyte populations among various treatment groups each assayed in triplicates±S.E.M.; *n*=3.

Caspases promote cell death in response to proapoptotic signals, and activation of caspase-3/7 is a hallmark characteristic of classic apoptosis. Thus, it was not surprising that the percentage of spermatocytes expressing activated caspase-3/7 significantly increased to ~68% upon CSC exposure for 18 h. Next, we assessed the role of AHR in expression of caspase-3/7 by treating cells with AHR-inh, which we confirmed did not on its own increase the number of caspase-3/7-positive cells (Supplementary. Figure 1A and B). Although pretreatment with AHR-inh at low concentrations (10 and 25 *μ*M) did not prevent CSC-induced caspase-3/7 activation, concentrations at 50 *μ*M and above significantly reduced the percentage of caspase-3/7-positive spermatocytes (Figures 5a and b). By contrast, siRNA-mediated knockdown of *Ahr* did not reduce the percentage of CSC-induced caspase-3/7-positive spermatocytes (Figures 5c and d). Likewise, WT and AHR-KO MEFs treated with CSC had equivalent percentages of caspase-3/7-positive cells (Figures 5e and f). These data indicate that, although CSC-mediated caspase activation occurred in the absence of AHR, blocking AHR activation with an inhibitor blunted CSC-induced caspase activation.

### AHR modulates CSC-induced cell membrane damage during apoptosis

**Figure 6:**
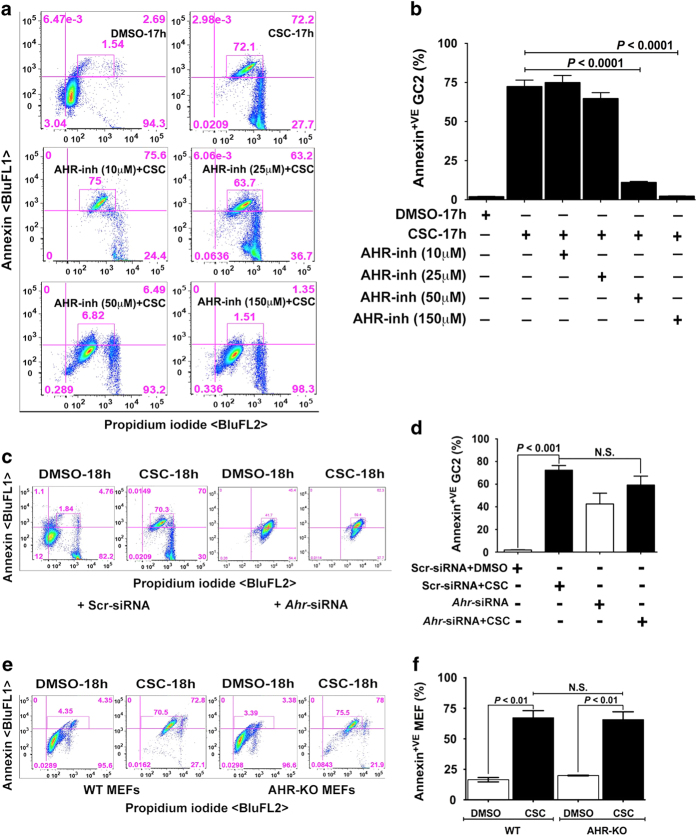
AHR-antagonist prevents CSC-induced cell membrane damage during apoptosis. **a**, **c** and **e**). The flow cytometric data represent the distribution of GC-2spd(ts) cells (**a**) treated with DMSO or CSC (40 *μ*g/ml) for 17 h, AHR-inh alone at different concentrations (10, 25, 50, and 150 *μ*M) or AHR-inh for 1 h followed by CSC, spermatocytes transfected with scr-siRNA or *Ahr*-siRNAs and (**c**) WT and AHR-KO MEF cells (**e**) treated with DMSO or CSC for 18 h. The treated spermatocytes were labeled with green annexin V Alexa Fluor 488 conjugate and counter-stained with PI for detecting externalization of phosphatidylserine. Following staining, the cell specimens of various groups were evaluated for positive cell populations based on standard compensation calculations on a BD FACSCalibur using FlowJo software (v9.7.5, FLOWJO, LLC.). The percent difference in annexin V^+^ cells is shown within the quadrant. **b**, **d** and **f**). Histogram represents the mean flow cytometric data of percent of annexin V and PI^+^ (BluFL1 and BLuFL2 double-positive) spermatocyte populations among various treatment groups each assayed in triplicate±S.E.M.; *n*=6.

We next asked whether CSC could also induce structural damage to the spermatocyte cell membrane. Thus, we used annexin V staining as a marker for externalization of phosphatidylserine. As seen in Figures 6a and b, about 70% of spermatocytes exposed to CSC were positive for annexin V. We next pretreated cells with the AHR-inh, which we confirmed did not cause an increase in the number of annexin-positive spermatocytes (Supplementary. Figure 2), and found that high doses (50 *μ*M and 150 *μ*M) significantly reduced the percentage of annexin-V-positive spermatocytes after CSC treatment (Figures 6a and b). However, siRNA-mediated knockdown of *Ahr* did not reduce the percentage of CSC-exposed annexin V-positive cells (Figures 6c and d). Likewise, WT and AHR-KO MEFs treated with CSC had equivalent percentages of annexin-V-positive cells (Figures 6e and f). These data indicate that CSC structurally modifies the spermatocyte cell membrane during apoptosis, and that, whereas this event is not affected by loss of AHR expression, it is abrogated by inactivation of the AHR pathway.

### Blocking activation of MAPKs does not prevent CSC-induced membrane damage

**Figure 7:**
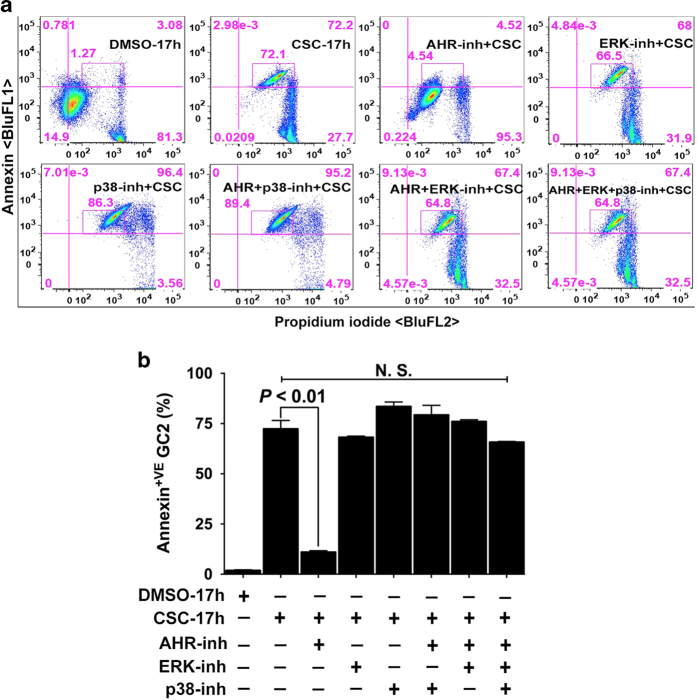
Inactivation of MAPKs does not prevent CSC-induced membrane damage. (**a** and **b**). Spermatocytes were exposed to DMSO (0.1%) or CSC (40 *μ*g/ml) for 17 h. In the antagonists treatment groups, the cells were first pretreated with AHR or MAPK inhibitors or together for 1 h followed by exposure to CSC for 17 h (**a**). The percentage of cells segregated following co-staining with annexin V alexa fluor 488 and PI was determined by FACS analysis using FlowJo software (v9.7.5, FLOWJO, LLC.) as described under materials and methods. The representative histogram demonstrates the distribution of spermatocytes at 17 h. The percent difference in annexin V^+^ cells is shown within the quadrant. (**b**) Histograms represent the mean flow cytometric data from DMSO-, CSC- or antagonists pretreated samples at 17 h of more than three independent experiments, each assayed in triplicate±S.E.M. *n*=4.

Given our previous study demonstrating crosstalk between p38-MAPK, ERK-44/42 and AHR,^19^ we asked whether pharmacological inhibition of MAPKs could prevent CSC-induced membrane damage. We first confirmed that neither the p38 inhibitor SB203580 nor the ERK inhibitor PD98059 could, on their own, increase the percentage of annexin-V-positive spermatocytes (Supplementary Figure 3). Pretreatment with MAPK inhibitors alone or together with AHR-inh could not prevent the externalization of phosphatidylserine in spermatocytes and thus the membrane damage (Figures 7a and b). These data ruled out the possibility of direct participation of MAPKs in membrane damage while implicating the specificity of AHR pathway in apoptosis.

## Discussion

In this study, we tested the hypothesis that inactivation of AHR could prevent CSC-mediated cell death in the spermatocyte cell line GC-2spd(ts). Our data demonstrate that CSC exposure activated intrinsic pathway of apoptosis via both AHR-dependent and -independent mechanisms. These studies revealed differential expression and activation of antiapoptotic and proapoptotic effector molecules under *Ahr* suppressed and *Ahr*-null conditions. Our approach adapting pharmacological intervention of AHR cascade by using CH223191 substantially decreased the susceptibility of CSC-induced GC-2spd(ts) from initiating apoptotic responses such as caspase activation, DNA damage and membrane alteration.

Fibroblasts and epithelial cells like GC-2spd(ts) are sensitive to the oxidative properties of CS. Our data demonstrating the generation of free oxygen radicals both in the cytoplasm and mitochondria of spermatocytes by the constituents of CSC complement our previous study^13^ and others.^20^ Mitochondrial hyperpolarization (Δψm) is an early apoptotic event and it has been shown that the activated AHR can regulate mitochondrial function through ATP5α1.^21^ In this study, the lack of change in Δψm by 4% CSC up to 6 h indicated that the magnitude of mitochondrial oxidative stress was not sufficient enough to alter the membrane potential at the given time point and concentration (40 *μ*g). We believe that the exposure of spermatocytes to 4% CSC for longer duration would alter the Δψm and this data correlates to several other studies of AHR ligands exposure in different cell types.^22^ As CSC has forced significant populations of spermatocytes into oxidative stress within 6 h and the mitochondria is the major intracellular source of ROS, we investigated the involvement of BCL2 family members. Various kinds of cellular stress lead to a shift in mitochondrial membrane potential and outer membrane permeabilization through activation of proapoptotic proteins of the BCL2 family. In contrast, the antiapoptotic members inhibit membrane permeabilization.^23^ In this study, the CSC-exposed spermatocytes under both normal and *Ahr*-silenced conditions, as well as the WT and AHR-KO MEF expressed equivalent levels of BCL2L1 and BCL2 proteins, corroborating with a previous study in BCL2L1 expression.^24^ These findings suggest that these prosurvival proteins are not under the direct regulation of AHR and their elevation might be an adaptive mechanism for cell survival against growth-inhibitory CSC.

Apoptosis is a highly balanced process between proapoptotic and antiapoptotic genes and AHR coordinates between cell proliferation and cell death.^15^ Furthermore, stable AHR KD markedly inhibited growth and promoted apoptosis.^25^ Several exogenous AHR ligands including benzo[a]pyrene in Hepa 1c1c7 cells^26^ and dimethylbenz[a]anthracene in preB cells^27^ have been reported to cause apoptosis. In this study, the CSC-induced increase in proapoptotic BAX^+^ and BAD^+^ populations independent of AHR, while in agreement to a previous study by Goode *et al.*,^28^ is contrary to an earlier report demonstrating the direct role of AHR in BAX expression in mouse oocytes.^29^ This discrepancy in regulation can be explained by the observation that the responses to apoptotic stimuli appear to be cell type-and dose-dependent because of the varied expressions of these complex pathways^30^ and the involvement of oxidative stress in AHR-deficient cells^31^ rather being solely mediated by AHR.

Our previous studies and others have shown that the constituents of CSC either directly or indirectly regulate genes associated to DNA damage and are responsible for causing DNA strand breaks in spermatocytes under both *in vitro* and *in vivo* conditions.^13,32^ We show here that the pretreatment with CH223191 but not the siRNA-mediated *Ahr* silencing significantly reduced the temporal increase in CSC-induced DNA strand breaks and the *Ahr* suppression alone has significantly elevated the TUNEL^+^ population of spermatocytes. These data suggest that the AHR might have a role in regulating the genes involved in the early stages of DNA damage such as *Cyp1a1*, *p21* or repair mechanism as demonstrated in our earlier studies^13,33^ and loss of AHR itself might trigger the signal for DNA damage as reported earlier.^34^ PARP acts as nick sensor during DNA repair and apoptosis^35^ and its cleavage serves as a hallmark of classical apoptosis and caspase activation. We observed in this study that the percentage of spermatocytes expressing cleaved PARP was marginal though significant and neither *Ahr*-silencing nor CH223191 could prevent the CSC-mediated PARP cleavage. However, the marked elevation in CSC-induced PARP^+^ populations among *Ahr*-suppressed spermatocytes coincides with previous studies demonstrating increased levels of cleaved PARP in AHR-KD epithelial cells.^34,36^

Caspase-3 is a frequently activated protease indispensable for apoptotic chromatin condensation and DNA fragmentation.^37^ The effect of CS on caspase-3 activity differs significantly among various cell types.^38^ In this study, the marked increase in CSC-induced caspase-3/7^+^ spermatocytes agrees with an earlier observation,^39^ but differs from others.^40^ This divergence in caspase-3 activation by CSC can be attributed to the heightened sensitivity of this enzyme to ROS accumulation by high concentration of CSC.^41^ Meanwhile, the dose-dependent inhibition of caspase-3/7 activity by CH223191 at higher concentrations (>50 *μ*M) suggesting the role of AHR and its failure to prevent caspase activation at low dosage is in agreement to an earlier report.^42^ Our effort to further understand AHR regulation of caspase-3 through siRNA silencing and AHR-KO MEF reveals that the lack of AHR increased basal activity in knockdown spermatocytes, while the addition of CSC might augment caspase activation due to oxidative stress. Our data contradicts previous studies by showing the deficiency in AHR leading to mitochondrial dysfunction and caspase-3 activation^43,44^ This finding can be explained, however, by the fact that the cells become more susceptible to apoptosis in absence of AHR^45^ and in the presence of stimuli of the intrinsic pathway.^16^

Cigarette smoking deteriorates the plasma membrane integrity of sperm cells.^46^ Externalization of phosphatidylserine as detected by annexin V staining is a sensitive, and quantitative approach in apoptosis. Our data here demonstrating the externalization of phosphatidylserine following CSC exposure has been reported in various other cell types.^38,47^ AHR targeted pharmaceuticals are AHR-specific regulators and their antagonistic activities are structure, cell context, response and dose dependent.^48^ As noticed, the cell membrane protective ability of CH223191 at higher dosage and the maximum amount of apoptotic populations in *Ahr*-deficient conditions suggests a cytoprotective role of AHR as suggested in other studies.^49^ Our observation is in accordance to a previous study by Rico de Souza *et al.* (2011)^36^ which demonstrates increased sensitivity of AHR-deficient cells due to reduced expression of super oxide dismutase. In addition, the ability of AHR to regulate apoptosis seems to be a universal phenomenon as AHR-suppressed spermatocytes also displayed increased sensitivity to smoke-induced apoptosis. Even though the pharmacological inhibition of CSC-activated MAPKs abrogates cell cycle arrest,^19^ the failure of MAPK-inh alone or together with CH223191 in attenuating the CSC-induced membrane alteration in spermatocytes is attributed to the complex free oxygen radicals of CSC^50^ and the involvement of other signaling mediators.^51^

Thus, the AHR antagonism using inhibitors such as CH223191 may represent a viable prophylactic therapeutic strategy in the prevention of cell death mediated by CS exposure and other similar fuel burning derived pollutants. The data of the current proposal becomes highly relevant by vividly highlighting the difference between the cell types towards a common physiological process via its molecular response to CS and the molecular divergence of pharmacological inhibition and genetic manipulation. This study collectively suggests that AHR is a viable therapeutic target to prevent CS-induced germ cell death.

## Materials and methods

### Cell culture and *in vitro* CSC and antagonists treatment

The mouse spermatocyte cell line GC-2spd(ts) (ATCC, Manassas, VA, USA),^52^ hereafter referred to as spermatocytes, was grown to 70% confluence and then serum starved for 24 h. Then, the growth-synchronized cells were treated with 40 *μ*g/ml CS condensate (CSC; in 100% dimethyl sulfoxide (DMSO); Murty Pharmaceuticals Inc., Lexington, KY, USA; prepared as detailed in our previous study^31^) or 0.1% DMSO for different time durations. In some cases, spermatocytes were pretreated for one hour with various concentrations of the AHR-specific antagonist CH223191 (2-Methyl-2H-pyrazole-3-carboxylic acid-(2-methyl-4-*o*-tolyl-azophenyl)-amide; referred to herein as AHR-inh; EMD chemicals, Gibbstown, USA).^42,53^ The p38 MAPK inhibitor SB203580 (EMD chemicals, Gibbstown, USA)^54^ was used at 10 *μ*M, and the ERK-MAPK/MEK1 inhibitor PD98059 (EMD chemicals, Gibbstown, NJ, USA)^55^ was used at 25 *μ*M/ml.

### Animals and MEF isolation

C57BL/6J WT and AHR-knock out (AHR-KO, B6.129-Ahr^tm1Bra^/J; The Jackson lab, Bar Harbor, ME, USA) mice were housed, bred, and treated experimentally in accordance with the NIH guidelines for the humane and ethical treatment of animals. All studies were approved by the Animal Studies Committees at Washington University School of Medicine and the St. Louis VA Medical Center. MEFs were isolated as described^40^ from e10.5 embryos and cultured after confirmation of their genotypes.

### SiRNA transfection

Spermatocytes were transfected with siRNAs as previously described.^55^ In brief, spermatocytes grown in 12-well plates were transfected overnight with siRNAs to *Ahr* or scrambled (scr) siRNAs (Ambion, Carlsbad, CA, USA) at a final concentration of 140 nM in Dulbecco’s Modified Eagle Medium. Cells were then treated as described for each experiment and analyzed by flow cytometry with appropriate antibodies. Each transfection assay was repeated a minimum of three times, and the results are shown as the mean±S.E.M. of independent experiments.

### Flow cytometry and analysis

Spermatocytes (1x10^6^/ml) exposed to CSC and various inhibitors as described in Results were analyzed on a FACScalibur flow cytometer (Becton-Dickinson, MountainView, CA, USA) as previously described.^19^ FlowJo software (v9.7.5, FLOWJO, LLC.) was used for analysis. To analyze expression patterns, a gate was first drawn around the single-cell populations in a dot plot of forward scatter *versus* side scatter. A dot plot of BluFL1 *versus* TO-PRO-3 (RedFL1) / PI (BluFL2) / SYBR 14 (BluFL4) was drawn according to the subclass control, and the quadrant markers were set according to both the vehicle (DMSO) control and the isotype control. The percent of protein/probe-positive (probe^+^) spermatocytes was computed by using the following formula based on the percentage of gated values for the BluFL1^+^ cells: (stimulated − stimulated isotype control) − (unstimulated − unstimulated isotype control). For probes that shift in fluorescence, experimental cells were standardized to vehicle-treated cells.

### Determination of oxidative stress in GC-2spd(ts)

Oxidative stress was detected by using CellROX deep red reagent and MitoSOX red mitochondrial superoxide indicator (both from Molecular Probes, Eugene, MN, USA). CellROX exhibits fluorescence (emission maxima at 650 nm) upon oxidation by reactive oxygen species, and MitoSOX red (emission maxima at 580 nm) detects superoxide in the mitochondria of live cells. Spermatocytes were treated with CSC or DMSO for 1 h and 5 h as previously reported.^13^ Then, CellROX and MitoSOX reagents were added to a final concentration of 1 *μ*M and 5 *μ*M, for 30 and 10 min, respectively, at 37 °C. The cells were washed with warm buffer as per the manufacturer’s protocol, counter-stained with SYBR 14 dye or TO-PRO-3 (Molecular Probes; 1 : 1,000) for 5 min at room temperature, and then subjected to flow cytometry.

### Evaluation of membrane potential change in mitochondria

Changes in the membrane potential of mitochondria were detected by using the MitoProbe DiOC2(3) Assay Kit (Molecular Probes) as per the manufacturer’s protocol. The GC-2spd(ts) cells exposed to CSC for 6 h were harvested, washed in warm PBS, incubated with a final concentration of 50 nM 3,3′-diethyloxacarbocyanine-iodide (DiOC_2_(3)) at 37 °C for 30 min, and then analyzed by flow cytometry. As a monomer, DiOC2(3) emits green fluorescence, and in a reaction driven by the mitochondrial membrane potential, it converts to a red-fluorescence–emitting dimer.

### Analysis of expression of BCL2 family proteins in spermatocytes

Spermatocytes transfected with scr-, or *Ahr*-siRNA were exposed to DMSO or CSC for 18 h and analyzed by flow cytometry. The following antibodies were used: rabbit anti-BCL2 (Abcam, Cambridge, UK), rabbit anti-BCL2L1 (Cell Signaling Technology) rabbit anti-BAX (Cell Signaling Technology, Boston, MA, USA), and mouse anti-BAD (Abcam, Cambridge, UK). Anti-BCL2 and anti-BAX rabbit primary antibodies were prelabeled by using zenon rabbit IgG labeling kits (Life technologies) as per the manufacturer’s protocol.

### Detection of DNA strand breaks in spermatocytes

To evaluate DNA damage, si-RNA-transfected cells were pretreated with 100 *μ*M AHR-inh for 1 h, then exposed to CSC or DMSO for 6 or 8 h. DNA fragmentation was detected by terminal deoxynucleotidyl transferase dUTP nick end labeling (TUNEL) as reported previously^56^ using the *in situ* cell death detection kit as per the manufacturer (Roche Diagnostics, Indianapolis, IN, USA). In other experiments, spermatocytes were pretreated with 100 *μ*M AHR-inh for 1 h, treated with CSC for 17 h, and stained with FITC mouse anti-cleaved Poly(ADP-ribose) polymerase (PARP) antibody (BD Biosciences, San Jose, CA, USA) and counter-stained with TO-PRO-3 (Molecular Probes) (1:1,000, 5 min) at room temperature. Cells were then analyzed by flow cytometry.

### Evaluation of caspase-3/7 activation in spermatocytes and MEFs

Spermatocytes were transfected with siRNAs or treated with 25, 50, 75, 100 and 150 *μ*M AHR-inh and then exposed to CSC for 18 h. WT and AHR-KO MEF primary cultures were similarly exposed to CSC. Following CSC treatment, the spermatocytes and MEFs were labeled by using cellEvent caspase-3/7 green flow cytometry assay kit (Molecular Probes) as per the manufacturer’s protocol and then analyzed by flow cytometry.

### Analysis of annexin V labeling in spermatocytes and MEFs

Spermatocytes were treated with DMSO or CSC for 17 h. Then cells were treated with 10, 25, 50, and 150 *μ*M AHR-inh 25 *μ*M ERK-inh, 10 *μ*M p38-inh, or a combination of all three inhibitors for 1 h. WT and AHR-KO MEF cultures exposed to CSC for 24 h, harvested, washed once with 0.1% BSA–PBS buffer, and then rinsed in 1X annexin binding buffer (0.1 M HEPES, pH 7.4; 1.4 M NaCl; 25 mM CaCl_2_), as described previously.^57^ Each 100 *μ*l of cell suspension was incubated with 2.5 *μ*l of FITC-conjugated annexin V (Molecular Probes), 1 mg/ml propidium iodide (Invitrogen, Carlsbad, CA, USA), and 50 *μ*g/ml RNaseA (Invitrogen) for 30 min at 37 ^°^C. Cells were then diluted into 500 *μ*l of 1× annexin binding buffer and analyzed by flow cytometry.

### Statistical analyses

Data are from three or four independent experiments; each assayed in duplicate or triplicate and represented as mean±S.E.M. The q-RTPCR data were analyzed by using either two-tailed unpaired *t*-tests or one-way ANOVA (nonparametric) followed by Tukey’s multiple comparison test with 95% confidence intervals. Prism 5.0d (GraphPad, La Jolla, CA, USA) was used, and *P*<0.05 was considered statistically significant.
