# Supplementary figures and images for: Cigarette smoke-induced cell death of a spermatocyte cell line can be prevented by inactivating the Aryl hydrocarbon receptor
Source: Cell Death Discov. 2015 Oct 26;1:15050–. doi: 10.1038/cddiscovery.2015.50 (PMC4979462; doi:10.1038/cddiscovery.2015.50)

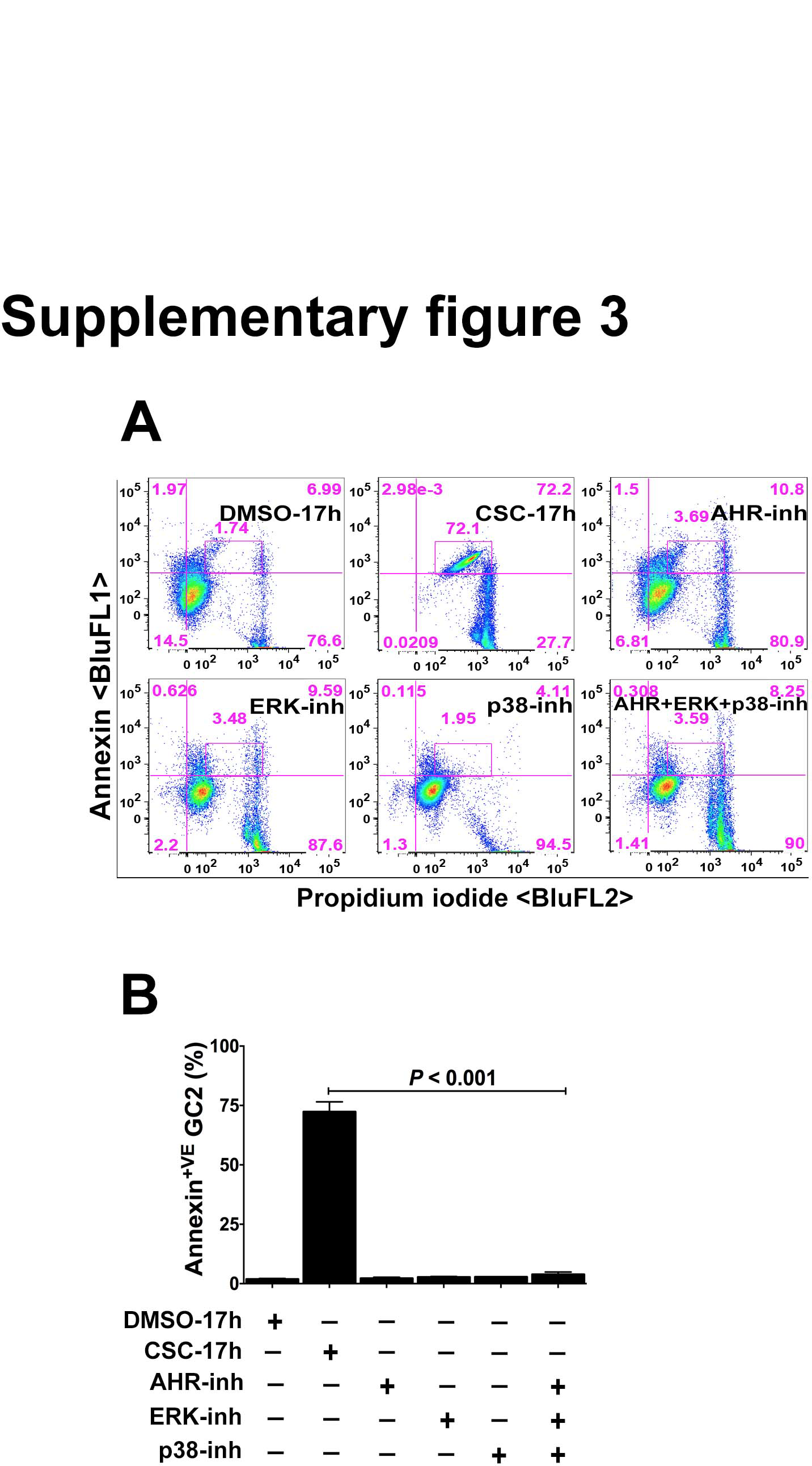

Supplement: Supplementary Figure 1 [file cddiscovery201550-s1.tiff]

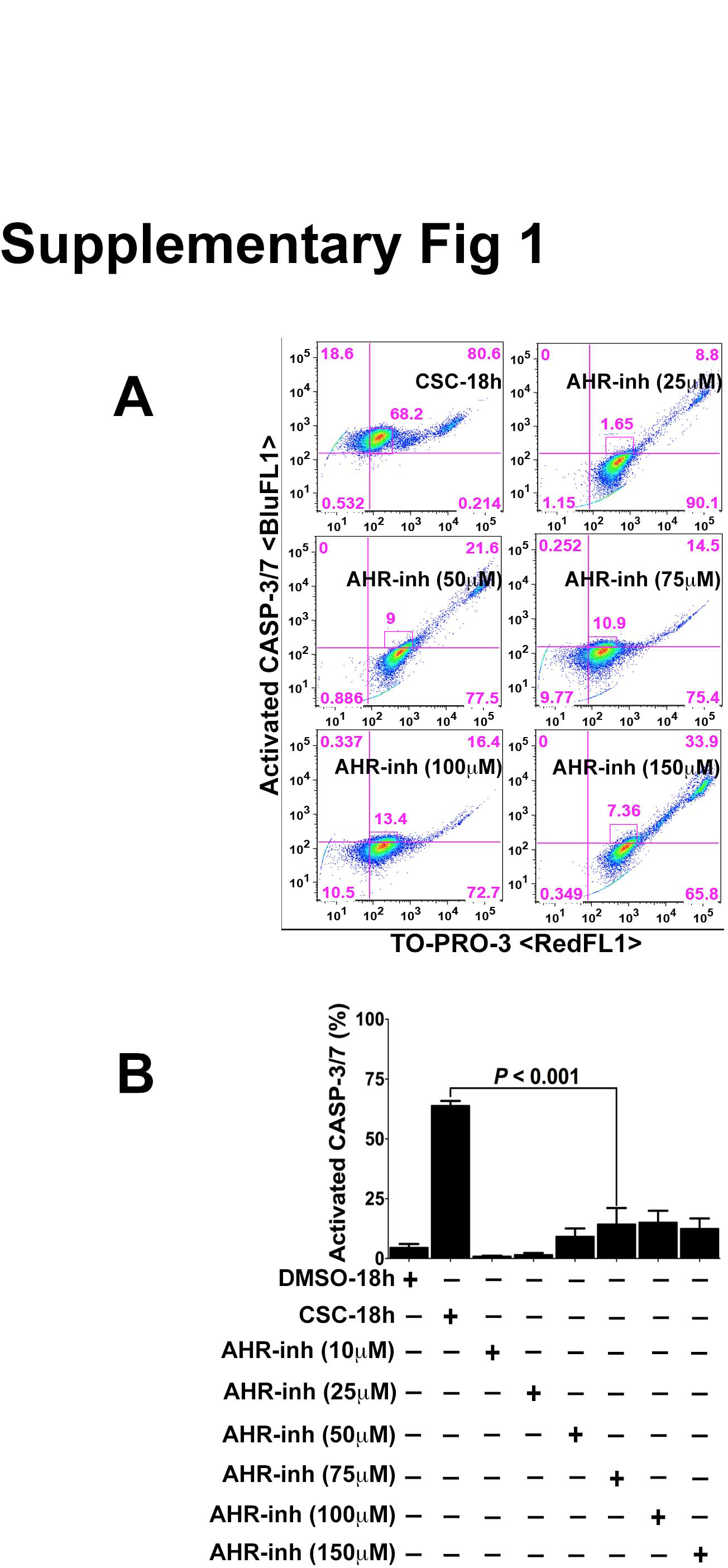

Supplement: Supplementary Figure 2 [file cddiscovery201550-s2.tiff]

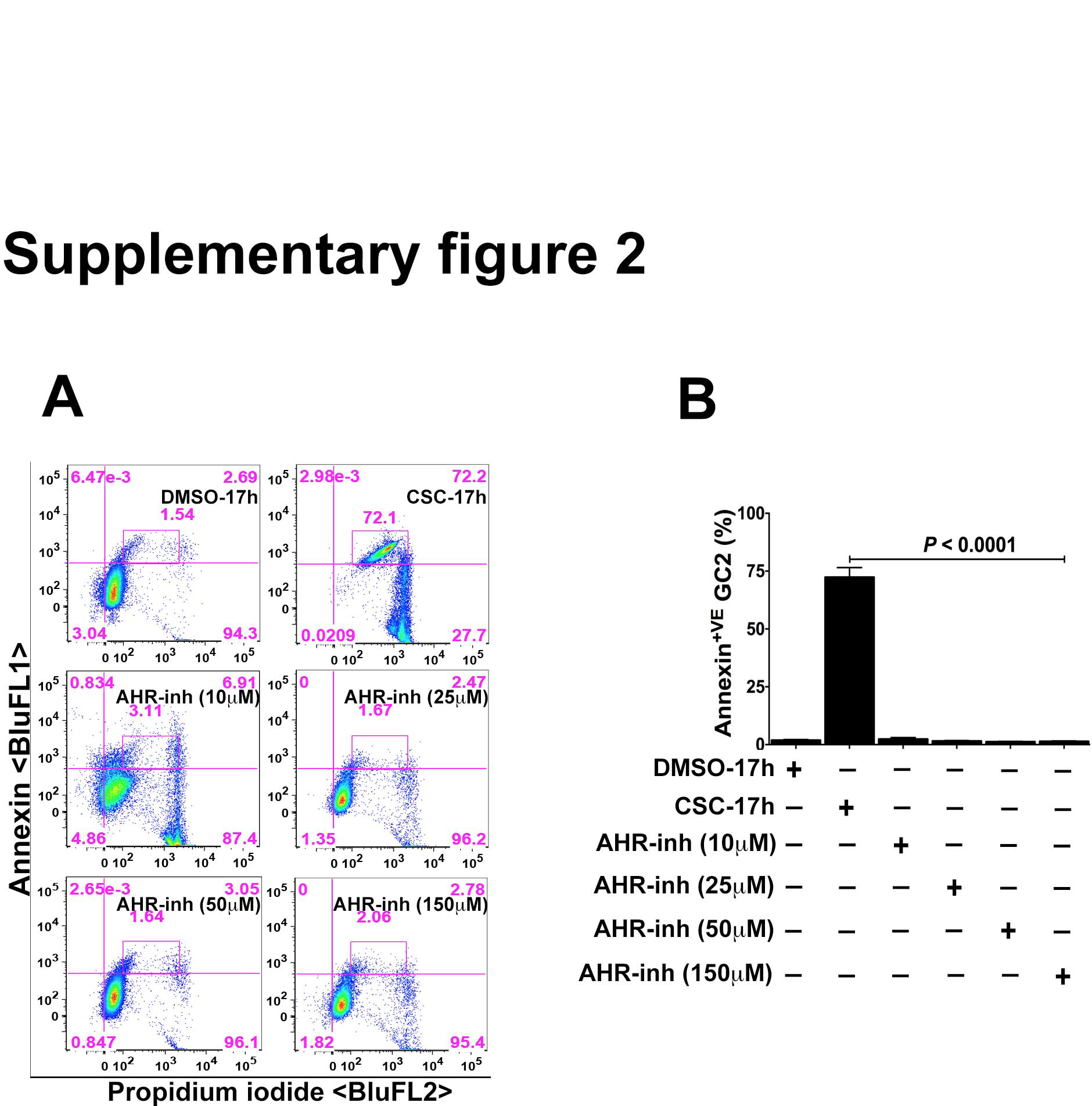

Supplement: Supplementary Figure 3 [file cddiscovery201550-s3.tiff]
