# Supplementary material for: Cigarette smoke-induced cell death of a spermatocyte cell line can be prevented by inactivating the Aryl hydrocarbon receptor
Source: Cell Death Discov. 2015 Oct 26;1:15050–. doi: 10.1038/cddiscovery.2015.50 (PMC4979462; doi:10.1038/cddiscovery.2015.50)
Supplement: Supplementary Information [file cddiscovery201550-s4.doc]

**Supplementary figures:**

**Fig 1, 2, and 3**: **Inhibitors itself are not agonists of apoptosis in spermatocytes.**

**1A, 2A,** and **3A).** To evaluate whether the AHR-, or MAPKs-inh alone or together activates caspase-3/7 (**1A**) or causes membrane damage to (**2A** and **3A**) spermatocytes, GC-2spd(ts) cells were exposed to different concentrations (25, 50, 75, 100, and 150 μM) of AHR-inh or MAPKs-inh and compared with DMSO (0.1%) or CSC (40 μg/ml) for caspase-3/7 activation (18h; **1A**) and annexin V staining (17h; **2A** and **3A**). The treated spermatocytes labeled with caspase-3/7 green detection reagent or green annexin V Alexa Fluor 488 conjugate and counterstained with TO-PRO-3 or PI were subjected to flow cytometry as described above. Data depict a representative result of three independent experiments and the percent difference in the generation of caspase-3/7^+VE^ and annexin V positive cells and percentages of cells are indicated within the quadrant. The subset populations within the quadrant were used for statistical analysis.

**1B, 2B,** and **3B).** Histogram represents the mean flow cytometric data of percent of BluFL1 and RedFL1 (**1B**) or BluFL1 and BluFL2 (**2B,** and **3B)** dual-positive spermatocytes from DMSO-, CSC-, and inhibitors treated samples of three independent experiments, each assayed in triplicates ± SEM.
